# Caffeine as an Ergogenic Aid for Neuromuscular Performance: Mechanisms of Action from Brain to Motor Units

**DOI:** 10.3390/nu18020252

**Published:** 2026-01-13

**Authors:** Paolo Amoruso, Edoardo Lecce, Alessandro Scotto di Palumbo, Massimo Sacchetti, Ilenia Bazzucchi

**Affiliations:** Laboratory of Exercise Physiology, Department of Movement, Human and Health Sciences, University of Rome “Foro Italico”, 00135 Rome, Italy; p.amoruso@studenti.uniroma4.it (P.A.); edoardo.lecce@uniroma4.it (E.L.); a.scottodipalumbo@uniroma4.it (A.S.d.P.); massimo.sacchetti@uniroma4.it (M.S.)

**Keywords:** caffeine, motor units, voluntary movement, ADORA2A, ergogenic aids, electromyography

## Abstract

Ergogenic aids have long attracted scientific interest for their potential to enhance neuromuscular performance, with caffeine being among the most extensively studied. While traditionally attributed to peripheral actions on skeletal muscle, accumulating evidence indicates that, at physiological doses, caffeine’s ergogenic effects are predominantly mediated by antagonism of central adenosine receptors. This antagonism leads to increased arousal, reduced inhibitory neuromodulation, enhanced corticospinal excitability, and altered motor unit recruitment and firing behavior. Importantly, the concentrations required to elicit direct effects on excitation–contraction coupling via ryanodine receptors exceed those compatible with human safety, rendering such mechanisms unlikely in vivo. This narrative review synthesizes contemporary neurophysiological evidence to propose that caffeine acts primarily by “tuning” motor system gain through central neurotransmitter modulation, rather than by directly augmenting muscle contractile properties. Additionally, we highlight unresolved questions regarding persistent inward currents, sex-dependent neuromodulatory influences—including the potential role of estrogen in regulating adenosine receptor expression—and the implications of repeated caffeine use during training for neural adaptation and motor control. Finally, we outline key methodological and conceptual directions for future research aimed at refining our understanding of caffeine’s neuromuscular effects in both acute and chronic contexts.

## 1. Introduction

Humans have sought external means to gain a competitive edge in sports since antiquity, with performance enhancement practices dating back to ancient Greece [1]. Ergogenic aids embrace a wide range of compounds that improve performance [2,3], and scientific advances have expanded the options available to athletes [3]. Among these, caffeine is one of the most well-established performance enhancers.

Caffeine (1,3,7-trimethylxanthine) is the most widely consumed psychoactive substance, with over 80% of the population using it regularly [2,4,5]. Since its removal from the World Anti-Doping Agency’s list of banned substances in 2004, reported use among athletes has substantially surged over the years [6,7]. Its ergogenic efficacy has been documented across multiple contexts, including aerobic [8] and anaerobic performance [9,10,11,12], in team sports [13,14,15], and for cognitive function [16].

The physiological basis of these improvements has been extensively investigated. Evidence points to four principal mechanisms: (1) modulation of ryanodine receptors, facilitating calcium ion release from the sarcoplasmic reticulum [17,18,19]; (2) inhibition of phosphodiesterases [2]; (3) antagonism of adenosine receptors [16,20]; and (4) regulation of extracellular potassium concentration through modulation of sodium/potassium pump activity [21]. However, calcium mobilization and phosphodiesterase inhibition require millimolar concentrations of caffeine [17,19,22], far exceeding the toxic threshold reported for humans (70 µM) [22]. Consequently, the ergogenic effects of caffeine at physiological doses are most likely mediated by its actions on the central nervous system (CNS) and on potassium permeability.

When examining the CNS, motor function is particularly relevant, as movement represents the primary means of interaction between the brain and the external environment. In particular, movement is achieved via spinal motor neuron activation of muscle fibers (i.e., motor unit [MU]), classically referred to as the *final common pathway* for motor control [23], which makes motor neurons central to studies of neuromuscular function. Advances in electromyographic (EMG) techniques, particularly high-density EMG (HDsEMG), now enable the extraction of detailed information regarding the neural commands transmitted to muscles [24], which gives insightful data about movement control [25]. Recent investigations have explored the hypothesis that caffeine may modulate MU behavior, providing intriguing evidence in this direction [26,27]. Nonetheless, available data remain limited.

Over the years, numerous reviews have examined specific aspects of caffeine’s effects, spanning from performance enhancement to neurophysiological outcomes. To date, only a few studies have summarized the evidence on caffeine’s effects on voluntary muscle activation [28,29,30]. However, these papers are relatively dated, highlighting the need for an updated synthesis that integrates recent findings. Accordingly, the purposes of this review are to: (a) summarize the most relevant and up-to-date evidence on the mechanisms by which caffeine facilitates voluntary muscle activation; (b) examine the current knowledge and remaining gaps regarding potential changes in MU behavior under caffeine consumption; (c) report the available findings on training regimens incorporating caffeine as a pre-workout strategy; and (d) propose an integrative theoretical framework and potential directions for future research.

## 2. Literature Search and Review Design

This manuscript was conceived as a structured narrative review rather than a formal systematic review. Accordingly, while a comprehensive and reproducible literature search strategy was employed, no quantitative synthesis, meta-analysis, or formal risk-of-bias assessment was undertaken.

An extensive literature search was conducted in PubMed/MEDLINE (National Center for Biotechnology Information, National Library of Medicine, National Institutes of Health, Bethesda, MD, USA), Scopus (Elsevier, Amsterdam, The Netherlands), and Web of Science (Clarivate Plc, Philadelphia, PA, USA) from database inception through October 2025. The search combined MeSH terms and free-text keywords designed to capture the full spectrum of caffeine-related mechanisms relevant to neuromuscular performance across multiple levels of the neuromuscular system, ranging from supraspinal and spinal processes to motor unit and peripheral adaptations. The primary search string was constructed as follows: (“caffeine” OR “methylxanthine”) AND (“neuromuscular adaptation” OR “neural adaptation” OR “brain/physiology” OR “spinal cord/physiology” OR “motor neurons/physiology” OR “motor unit” OR “motor unit recruitment” OR “corticospinal” OR “cortical plasticity” OR “spinal excitability” OR “synaptic plasticity” OR “persistent inward currents” OR “muscle hypertrophy” OR “peripheral adaptations” OR “hormonal response” OR “genes” OR “gene expression” OR “metabolism” OR “adenosine” OR “adenosine receptor” OR “ADORA2A” OR “CYP1A2” OR “myokines” OR “IL-6” OR “testosterone” OR “growth hormone” OR “sex differences”).

In addition to database searches, the reference lists of key original articles and recent reviews were manually screened to identify relevant studies that may not have been captured by the keyword strategy.

Eligible studies included original research articles and reviews investigating the effects of caffeine on central nervous system, spinal, motor neuron, motor unit, muscular, hormonal, metabolic, or molecular mechanisms with relevance to neuromuscular performance. Both human and animal studies were considered, provided that the findings offered mechanistic insight applicable to exercise performance. Studies were excluded if they were unrelated to neuromuscular function, focused solely on epidemiological associations without mechanistic relevance, or were non-peer-reviewed publications.

All identified records were screened by title and abstract, and full texts of potentially eligible articles were retrieved. In line with the narrative nature of the review, evidence was synthesized qualitatively. When findings were conflicting, greater emphasis was placed on studies with higher translational relevance (e.g., human experimental studies over animal models) and on convergent evidence across multiple methodological approaches. Discrepancies in the literature were explicitly discussed rather than resolved through quantitative weighting.

## 3. Caffeine Metabolization

Caffeine, a methylxanthine derivative (1,3,7-trimethylxanthine) (for detailed review, see Reddy et al. [31]), is among the most efficiently absorbed ergogenic substances, with a bioavailability approaching 100% [32]. Following oral ingestion, caffeine is rapidly absorbed from the gastrointestinal tract [33] and, owing to its lipophilic nature, promptly crosses biological membranes, including the blood–brain barrier [34]. Caffeine appears in the bloodstream shortly after oral ingestion, typically reaching peak plasma concentrations within one hour [35]. In adults, the half-life of caffeine exhibits considerable inter-individual variability that is influenced by multiple factors, such as genetic predisposition, smoking, biological sex, pregnancy, and the use of oral contraceptives [36,37]. Caffeine and its metabolites are excreted mainly in the urine [31], which constitutes a non-invasive, reliable means to detect its consumption in sports medicine [6].

### 3.1. Genetic Interindividual Differences in Caffeine Metabolism and Responsivity

The genetic information encoding the enzymes responsible for caffeine metabolism is partly inherited [36]. After absorption, caffeine is predominantly metabolized in the liver by cytochrome P450 1A2 (CYP1A2) enzyme [38]. Based on *CYP1A2* genotype, individuals are commonly classified into two categories according to their metabolic rate: carriers of the *AC* or *CC* genotypes are considered slow metabolizers, whereas *AA* carriers are classified as fast metabolizers (see Guest et al. [39] for details).

Another key genetic moderator of caffeine’s ergogenic effect is the *ADORA2A* gene variation, which encodes the adenosine A2A receptor. Caffeine’s ergogenic effects are largely attributed to the antagonizing action on this receptor [20], to which caffeine shows higher affinity compared with A1 receptors [40,41]. Consequently, polymorphisms in the *ADORA2A* gene significantly affect individual responsiveness to caffeine. Indeed, a study by Loy et al. [42] reported greater sensitivity in individuals carrying the *TT* allele compared with *CT/CC* carriers. Interestingly, genotypic differences also influence the hormonal response to strenuous exercise, with *TT* carriers exhibiting higher serum levels of growth hormone and testosterone [43].

### 3.2. Systemic Responses to Caffeine Ingestion

Caffeine can increase catecholamine turnover through both central and peripheral mechanisms [44]. Earlier research proposed that methylxanthines may induce norepinephrine release (see Table 1 for a summary) directly from sympathetic nerve terminals via antagonism of A1 receptors [45]. However, norepinephrine release is primarily regulated by presynaptic α2-receptors rather than adenosine-mediated mechanisms [46]. Therefore, the main origin of heightened norepinephrine levels appears to be caffeine’s direct action on the brain’s sympathetic control centers [47]. Indeed, methylxanthines have been shown to increase firing rates of noradrenergic neurons within the locus coeruleus [48]. Elevated levels of circulating norepinephrine increase arousal [46] and exert both chronotropic and inotropic effects on cardiac muscle [49]. Consequently, caffeine promotes the increase in heart rate both indirectly (i.e., norepinephrine release) and directly by blocking adenosine receptors in pacemaker cells [50].

Additionally, caffeine has been suggested to enhance lipolysis through direct stimulation of the adrenal medulla, thereby promoting epinephrine release [51]. Moderate to high doses of caffeine are associated with significant increases in circulating catecholamines and free fatty acids [52]. Nevertheless, such alterations in circulating compounds do not appear to yield any significant improvement in endurance performance compared with placebo [52], thereby supporting the predominance of caffeine’s central mechanism of action.

During and after intense physical activity, numerous signaling hormones are released into the bloodstream to either acutely enhance performance (e.g., catabolic pathways) or facilitate post-exercise recovery and tissue remodeling (e.g., anabolic pathways). In this context, testosterone and growth hormone are key mediators in these anabolic processes. Evidence indicates that caffeine consumption can influence testosterone and growth hormone release both during [53] and after intense exercise [43]. Specifically, Beaven et al. [53] reported a small but significant dose-dependent increase in testosterone levels during exercise following caffeine ingestion, up to a dose of 800 mg, accompanied by a concomitant rise in cortisol levels that potentially attenuates the net anabolic response. Similarly, Rahimi et al. [43] observed significant post-exercise increases in serum growth hormone and testosterone following a single bout of strenuous activity. Notably, this effect was statistically significant only in individuals carrying the *TT* allele, thereby supporting genotype-dependent variability in response to caffeine administration [42].

## 4. Modulation of Central Activity

**Figure 1:**
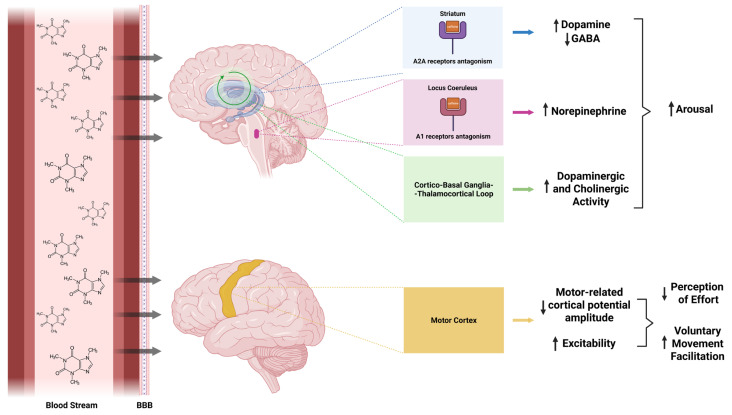
Theoretical graphical representation of putative central mechanisms through which caffeine may modulate voluntary movement, based on a synthesis of animal data, pharmacological evidence, and indirect human observations. “↑” indicates an increase in the measured activity, release, or parameter, whereas “↓” indicates a decrease in the measured activity, release, or parameter; the circular green arrow represents the cortico–basal ganglia–thalamocortical loop; BBB = Blood–Brain Barrier. Created in Biorender. Paolo Amoruso. (2025), https://app.biorender.com/illustrations/68fb34a422db4b4755a06954.

At physiological doses, caffeine primarily targets the CNS, Within the brain, caffeine modulates multiple, partially overlapping processes that support voluntary motor performance [28,29]. Although these processes are closely interrelated, they are discussed separately here to distinguish state-dependent effects (*arousal*), signal-level modulation (*corticospinal modulation*), and perceptual consequences (*perception of effort*). Lastly, Figure 1 presents a theoretical graphical representation integrating the putative mechanisms of action of caffeine in the brain.

### 4.1. Arousal

Voluntary movement and neuromuscular performance depend on an optimal level of cortical readiness, whereby the CNS is sufficiently activated to support higher-order motor behaviors [54,55]. This relationship is well established in sports psychology and is commonly described by an inverted-U association between arousal and performance [56]. Neurophysiologically, arousal can be assessed using skin conductance level (SCL) or electroencephalographic (EEG) alpha activity [57]. These measures are closely related, as reductions in alpha power and concomitant increases in alpha frequency correspond to elevations in SCL [58]. Consistent with caffeine’s stimulatory properties, experimental studies have reported increased SCL following ingestion of 250 mg of caffeine [58] as well as suppression of EEG alpha power after a lower 50 mg dose [59].

Using advanced fMRI, Singh and colleagues [60] further characterized the neural substrates linking arousal to motor control, providing insight into how caffeine may facilitate movement indirectly through arousal-related networks. Specifically, caffeine disinhibits striatal neurons via antagonism of adenosine A2A receptors, enhancing dopaminergic signaling and reducing inhibitory GABAergic output from the striatum. This mechanism facilitates thalamocortical activation and supports movement initiation [30].

In parallel, caffeine disinhibits key arousal centers within the pontine and mesencephalic reticular formations, including the locus coeruleus [48]. Activation of these structures likely increases noradrenergic drive to widespread cortical and subcortical regions, thereby establishing a neurophysiological state permissive to efficient motor execution. Additionally, caffeine enhances activity within the cortico–basal ganglia–thalamocortical loop by amplifying dopaminergic and cholinergic tone (see Fisone et al. [30] and Ferré [61]), further supporting motor readiness and action selection.

### 4.2. Corticospinal Modulation

Beyond its role in regulating arousal, caffeine also influences cortical activity and information processing. Hemodynamic studies provide important insight into this phenomenon. Xu et al. [62] reported a marked reduction in cerebral blood flow following ingestion of 200 mg of caffeine; however, this effect was accompanied by improved oxygen extraction efficiency and increased neuronal activity. Subsequent investigations have corroborated these findings, demonstrating reduced prefrontal cortex oxygenation after caffeine doses of 200 mg [63] and 6 mg·kg^−1^ [64]. Notably, in the latter study, reduced prefrontal oxygenation coincided with decreased motor cortex activation during exercise, suggesting more efficient neural processing rather than impaired cortical function [64]. Consistent results were also reported by Chang et al. [65] who observed reduced prefrontal blood flow alongside increased brain entropy, indicative of enhanced information-processing capacity. These hemodynamic effects are consistent with caffeine’s antagonistic action on adenosine receptors, whose activation normally promotes vasodilation and suppresses neural activity [66].

Caffeine has the potential to shift cortical networks toward increased excitability and efficiency. Early animal studies demonstrated enhanced cortical electrical activity following caffeine administration [67,68] and more recent human investigations have extended these findings using transcranial magnetic stimulation (TMS). In humans, ingestion of 200 mg of caffeine has been shown to amplify neural responses to TMS, indicating that a lower external stimulus is required to evoke comparable corticospinal output [69]. Similarly, Cerqueira et al. [70], reported reductions in cortical silent period (CSP) duration following the same dose, consistent with increased central excitability. Comparable effects have been observed using a relative dose of 6 mg·kg^−1^, including reductions in short-interval intracortical inhibition [71]. However, interpretation of CSP remains challenging, as this measure reflects contributions from both cortical and supraspinal sources [72], and not all studies have reported consistent reductions in CSP duration [73,74].

Importantly, even in the absence of clear changes in cortical inhibition, caffeine has been shown to enhance corticospinal output under specific conditions. For example, Bowtell et al. [74] reported greater motor evoked potential (MEP) amplitudes during high-intensity contractions following ingestion of 6 mg·kg^−1^ of caffeine, while Kalmar and Cafarelli [75] observed increased MEP amplitudes at the onset of fatiguing exercise but not at rest. These findings are consistent with post-exercise facilitation phenomena, which can also occur in the absence of caffeine following voluntary contractions [76].

Overall, these data suggest that caffeine’s effects on cortical function are best characterized as task- and state-dependent enhancements in corticospinal transmission, rather than uniform reductions in cortical inhibition or direct disinhibition of motor cortical circuits.

### 4.3. Perception of Effort

Beyond its influence on arousal and corticospinal excitability, caffeine also alters the conscious perception of effort, a key determinant of endurance and exercise tolerance. Perception of effort is commonly quantified using Borg’s Rating of Perceived Exertion (RPE) scale [77] and arises from the integration of afferent feedback with central motor command signals [78]. In addition to peripheral sensory input, the subjective experience of effort is strongly influenced by internal copies of motor commands generated within premotor and motor cortical regions during voluntary activation [78,79,80].

The motor-related cortical potential (MRCP) provides a neurophysiological index of cortical activity associated with movement preparation and execution. Supporting its relevance to effort perception, de Morree et al. [80] demonstrated a positive association between MRCP amplitude and RPE, suggesting that greater cortical engagement corresponds to higher perceived effort. Accordingly, reducing the neural resources required to sustain a given task intensity should improve performance capacity.

Caffeine appears to exert such an effect. Pires et al. [64] reported reduced motor cortex activation alongside improved intermittent exercise capacity following ingestion of 6 mg·kg^−1^ of caffeine. Using a similar dose, Mesquita et al. [71] observed increased time to task failure and lower RPE, accompanied by a shortened CSP, consistent with more efficient central motor command. Further supporting this interpretation, de Morree et al. [81] reported significantly lower MRCP amplitudes and RPE during intermittent isometric knee extensions following caffeine ingestion, despite comparable muscle activation and force output relative to control conditions.

Collectively, these findings provide a neurophysiological basis for the well-documented ergogenic effect of caffeine on perceived exertion [82,83,84,85,86,87,88,89,90] suggesting that caffeine enhances performance in part by reducing the central neural cost associated with voluntary force production.

### 4.4. Long-Term Potentiation and Motor Learning

Beyond its acute effects on arousal and corticospinal transmission, caffeine has also been hypothesized to influence synaptic plasticity and motor learning; however, evidence in humans is limited and inconsistent.

Repeated activation of synaptic transmission leads to a durable strengthening of synaptic efficacy, commonly referred to as long-term potentiation (LTP), which is widely regarded as a fundamental mechanism underlying learning and memory. In the context of motor control, LTP-like processes are thought to contribute to motor learning and skill acquisition. Given caffeine’s ability to enhance neuronal excitability and firing rates [47], it has been hypothesized that caffeine might influence synaptic plasticity; however, direct evidence supporting this mechanism in humans remains limited. Animal studies provide mechanistic insights into this possibility: in rodents, caffeine has been shown to enhance LTP and increase neuronal metabolic activity within the striatum [91], a key input nucleus of the basal ganglia involved in the selection and execution of motor programs [30]. While enhanced synaptic plasticity within this region could, in principle, facilitate motor learning, the translational relevance of these findings to human motor performance remains uncertain and requires direct experimental validation. In contrast, evidence from human studies is sparse and inconsistent. Investigations targeting the primary motor cortex have reported a reduction in LTP-like plasticity following acute caffeine ingestion (200 mg) [40], suggesting a potential attenuation of cortical plasticity. Conversely, Concerto et al. [92] observed enhanced LTP-like responses in the form of post-exercise facilitation; however, their protocol involved the consumption of an energy drink containing multiple neuroactive substances, precluding attribution of these effects specifically to caffeine. Moreover, behavioral studies examining motor learning, memory acquisition, and long-term retention in humans have generally failed to demonstrate consistent benefits following caffeine intake [16]. Notably, this contrasts with the more consistent evidence supporting caffeine’s acute effects on arousal, corticospinal transmission, and neuromuscular performance.

Taken together, current evidence suggests that while caffeine reliably enhances acute neuronal excitability and motor performance, its effects on synaptic plasticity and motor learning remain equivocal. Consequently, any proposed role of caffeine in modulating LTP or long-term motor learning should be regarded as speculative at present, highlighting the need for carefully controlled human studies to clarify these mechanisms.

## 5. Modulation of Spinal Excitability

The means by which neural inputs from the CNS are translated into neural drive are the α-motor neurons [25,93]. The soma of these neurons is located within the spinal cord; therefore, modulation of synaptic transmission at this level strongly influences motor performance. Given the well-documented ergogenic effects of caffeine on various aspects of movement performance [10,11,39,94], it is pertinent to examine its effects on parameters of spinal excitability.

### 5.1. Hoffman Reflex

The monosynaptic Hoffmann reflex (H-reflex) is a commonly employed index of spinal excitability. It is analogous to the stretch reflex but bypasses muscle spindles [95], being elicited by a low-intensity, long-duration percutaneous electrical stimulus that preferentially activates large-diameter Ia afferent fibers projecting to the motor neuron pool [28]. Traditionally, spinal excitability has been quantified through the maximal amplitude of the reflex (H_max_). However, this measure may be insensitive to subtle excitability changes because it is constrained by geometric factors and saturation of the motor neuron pool [96]. The H-slope (H_slp_) has been proposed as a more reliable alternative [96]. This parameter reflects the slope of the ascending limb of the recruitment curve and indicates how rapidly the motor neuron pool is recruited, making it more representative of small changes in excitability [96].

Studies examining caffeine’s effects at the spinal level have produced equivocal findings. Some report increased spinal excitability [97], whereas others have found no significant changes [71,98,99,100]. For instance, Walton et al. [97] found H_slp_ to be steeper following a caffeine dose of 6 mg·kg^−1^, suggesting facilitated recruitment of the motor neuron pool. In contrast, Kalmar and Cafarelli [100] observed unchanged H_max_ amplitude in the soleus muscle following a similar dose of caffeine. Subsequent research corroborated this finding on multiple occasions [71,98,99]. Recently, Mesquita et al. [71] proposed that H-reflex measures may conflate presynaptic (Ia terminal) gating and postsynaptic motor neuron excitability, which complicates attribution of any caffeine effect to a specific spinal locus. Consequently, the authors suggested that cervicomedullary motor-evoked potentials (CMEPs) may represent a more appropriate approach for probing spinal excitability in caffeine research. Unlike the Ia-mediated H-reflex, CMEPs are elicited via activation of descending corticospinal axons and not influenced by the presynaptic inhibition that modulates Ia afferent terminals [101], making CMEP amplitude or threshold a more direct index of postsynaptic motor neuron excitability. Existing literature on this topic remains limited.

### 5.2. Intrinsic Motor Neuron Properties

Adult motor neurons are capable of sustaining repetitive firing through persistent inward currents (PIC), primarily generated by the prolonged activation of voltage-gated Ca2+ channels [102]. PICs amplify transient excitatory inputs and are therefore essential in the generation and maintenance of purposeful movements [25]. Importantly, PICs are dynamically modulated by descending monoaminergic drive, primarily noradrenaline and serotonin, originating from supraspinal centers [102]. Because direct in vivo assessment of PIC amplitude within the human spinal cord is not feasible, electromyographic techniques are typically employed to estimate their strength [103]. However, these approaches provide only indirect indices of PIC magnitude and rely on several assumptions, including relatively constant synaptic drive to the motor neuron pool, stable motor unit pairing, and consistent discharge behavior [25,103,104,105,106]. These assumptions may be violated under conditions of altered neuromodulatory state, such as following caffeine ingestion, potentially confounding the interpretation of estimated PIC-related metrics.

Caffeine has been shown to enhance the release of several neurotransmitters [47,107], including monoamines, by acting on brainstem nuclei such as the locus coeruleus (noradrenergic neurons) [48] and the raphe nuclei (serotonergic neurons) [108]. Notably, serotonergic projections from the raphe nuclei exert direct excitatory influences on spinal motor neurons [109]. Thus, caffeine could theoretically augment PIC amplitude indirectly through enhanced monoaminergic facilitation. Nevertheless, caffeine-induced changes in firing rate modulation, recruitment behavior, or descending synaptic input may also influence commonly used PIC estimates without necessarily reflecting true alterations in intrinsic motoneuronal properties.

Empirical findings on this matter are inconsistent. Some studies have reported increased self-sustained firing [97,110], whereas others have found no significant effects [27,111]. For instance, Walton et al. observed greater PIC amplitude [110] and enhanced plateau potentials [97] following ingestion of 6 mg·kg^−1^ of caffeine. In contrast, Kirk et al. [111] found no improvement using half that dosage. However, their evaluation of PICs was performed during non-voluntary contractions evoked through tendon vibration combined with electrical stimulation, thereby excluding contributions from descending voluntary drive, which can substantially influence PIC magnitude [102]. More recently, Mackay et al. [27] employed HDsEMG and a 6 mg·kg^−1^ dose but similarly reported no caffeine-induced enhancement of PIC amplitude during voluntary contractions. Taken together, these discrepancies may reflect not only differences in dosage, task characteristics, or muscle group examined, but also the limited sensitivity and assumption-dependence of current techniques used to estimate PICs in humans [102].

Collectively, available evidence suggests that caffeine’s facilitative effects on motor performance are more likely mediated by enhanced central command and corticospinal communication rather than by direct modulation of intrinsic motoneuronal properties. However, because previous investigations have predominantly examined lower-limb muscles, particularly the soleus [110,111] and tibialis anterior [27,97,111], and because PIC amplitude differs across muscles and experimental contexts [102], a potential indirect effect of caffeine on intrinsic motoneuronal properties cannot be entirely excluded. Further research employing refined methodological approaches is therefore warranted to clarify this possibility.

## 6. Acute Modulation of Motor Unit Behavior

The advent of HDsEMG has enabled a more precise investigation of electromyographic variables [112], allowing for the non-invasive characterization of MU behavior [24]. Because MU firing encodes the information sent from spinal and supraspinal areas, MU measures provide a direct window onto neural strategies for movement generation [24]. Given the central excitatory effect of caffeine, MUs represent a promising avenue for investigating its ergogenic action on movement generation.

Indirect measures of MU recruitment, such as the root mean square (RMS) value, reflect the average magnitude of muscle’s electrical signal over a specific time window. Consequently, RMS is influenced by the number of active MUs and their firing rate, particularly higher-threshold ones [113]. Caffeine ingestion has been shown to increase RMS values following a 6 mg·kg^−1^ dose [12]. Interestingly, the same authors also reported a decrease in muscle fiber conduction velocity [12]. Taken together, these findings suggest enhanced recruitment of higher-threshold MUs (HTMU) after caffeine consumption. However, other studies failed to observe similar increases in RMS [114,115].

**Figure 2:**
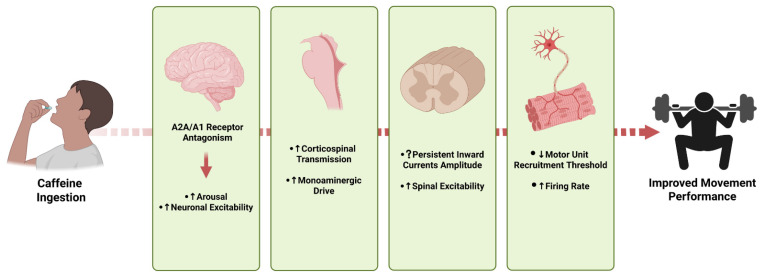
Simplified theoretical graphical representation of the areas involved in voluntary movement facilitation following the ingestion of caffeine. At physiological doses (≤70 µM), the ergogenic effect exerted by caffeine is most likely explainable by the tuning of the central nervous system towards a facilitation in neuronal firing rates, especially involving corticospinal communication. Any statement regarding caffeine-induced modulation of persistent inward currents should be interpreted with caution (indicated by the question mark symbol “?”), as current human evidence is conflicting and relies on indirect, assumption-dependent estimation methods. “↑” indicates an increase in the measured activity, release, or parameter, whereas “↓” indicates a decrease in the measured activity, release, or parameter. Created in Biorender. Paolo Amoruso. (2025). https://app.biorender.com/illustrations/6964c64cae2edc163fa79379.

Direct evidence on MU behavior is scarce, highlighting the need for further investigations. To date, only two studies have directly examined MU firing characteristics under acute caffeine ingestion using HDsEMG [26,27]. Mackay et al. [27] reported a decreased recruitment threshold (RT) at 20% of maximal voluntary force (MVF) in the tibialis anterior following caffeine intake (6 mg·kg^−1^), whereas a significant increase was observed at 40% MVF. Conversely, Nishikawa et al. [26] found different results in the vastus lateralis, where HTMU showed a reduced RT after ingestion of a fixed 200 mg dose of caffeine. Moreover, the same study reported an increase in firing rate among lower-threshold MUs (LTMU). A possible explanation for enhancements in firing rates among LTMUs might be the firing facilitation promoted by increased PIC amplitudes [102], crucial for low-intensity contractions and on which caffeine has the potential to act. Discrepancies between the findings reported by Mackay et al. [27] and Nishikawa et al. [26] may arise from differences in the muscles investigated (tibialis anterior vs. vastus lateralis) and caffeine dosages. Indeed, a more pronounced facilitatory effect has been reported in larger muscle groups [11], possibly due to differences in the excitation thresholds characteristic of HTMUs. It should be emphasized that much of the evidence informing the mechanistic interpretations linking MU behavior and movement per se derive from isometric contractions, where HDsEMG decomposition is most reliable. While these findings provide critical insight into neural control mechanisms [24], the direct translation of motor unit–level behavior to ballistic or highly dynamic movements remains largely theoretical, given current technical limitations in the field of electromyographic signal processing. Figure 2 provides a simplified theoretical representation of the areas involved in voluntary movement facilitation, summarizing the documented effects of caffeine ingestion discussed up to this point in the review.

### 6.1. Sex-Related Differences

Although evidence is limited, available data indicate that caffeine consumption amplifies neural drive, defined as the ensemble of action potential trains generated by the pool of motor neurons innervating a muscle, though this facilitation may differ between HTMU and LTMU. However, it remains unclear whether caffeine-induced enhancements in MU firing and excitability differ between males and females.

Investigations in female participants are inherently more complex due to additional variables that must be controlled, including menstrual cycle–related hormonal fluctuations and contraceptive use. For instance, menstrual cycle phase has been shown to influence the rate of caffeine metabolism [116], whereas oral contraceptives double the half-life of caffeine [36]. Interestingly, Skinner et al. [117] reported higher plasma caffeine concentrations in females compared with males following ingestion, suggesting slower metabolic clearance, despite similar performance improvements. As previously discussed, adenosine receptors mediate many of caffeine’s effects [46]. Females have been reported to exhibit higher A1 receptor densities in several brain regions [118], and estrogen has been shown to upregulate adenosine receptor expression in animal models [119]. Taken together, these findings suggest that hormonal modulation of adenosine receptor activity across the menstrual cycle could alter caffeine-induced enhancements in neuromuscular performance, potentially attenuating its facilitation effects on MU behavior in females relative to males.

### 6.2. Potential Sex-Related Differences in Acute Motor Unit Adaptations

Female individuals typically show higher firing rates in LTMUs [120,121,122,123,124], more frequent doublets [124] and larger estimates of PICs [125,126], suggesting a high baseline excitability at low forces. Consequently, the scope for further caffeine-induced facilitation of firing rate in LTMUs may be limited at matched relative intensities. Additionally, if females bias low-threshold drive at submaximal forces, caffeine’s most visible effect may instead be to disproportionately lower the recruitment thresholds of HTMUs as force demand increases, as generally observed by Nishikawa et al. [26]. In tibialis anterior tasks, where caffeine does not appear to alter PIC contribution in males [27], a similar null effect might therefore be expected in females, although other muscles could result in different outcomes. Finally, menstrual-cycle-related variability in MU behavior [127,128] could modulate the hypothesized adaptations to caffeine, shifting the locus of caffeine’s facilitation within the MU pool across phases, potentially enhancing recruitment during some phases and firing-rate modulation during others. These sex- and phase-contingent hypotheses warrant direct testing with MU-level tracking in females.

### 6.3. Potential Rate of Force Development Modulation

The rate of force development (RFD) is a critical determinant of performance across multiple settings [10,129,130,131]. However, evidence regarding the effects of caffeine on RFD remains inconsistent. While two studies reported no improvements in the biceps brachii [132] and soleus [71], only one study observed an enhancement in the vastus lateralis [133]. A recent meta-analysis nonetheless concluded that caffeine exerts a moderate positive effect on RFD [10], though the underlying neuromechanical mechanisms remain poorly understood.

Neural drive plays a pivotal role in determining RFD, particularly through modulation of MU firing rate and RT [134]. During rapid contractions, MUs fire up to three times faster than during sustained efforts and are recruited at comparatively lower force levels [133,134,135,136]. Simulation studies have demonstrated that increases in the firing rate of HTMUs directly enhance RFD [137]. Furthermore, serotonin has been shown to contribute to RFD modulation, as pharmacological blockade of 5-HT receptors reduces MU firing rates and consequently RFD [138]. Considering that caffeine can enhance neural drive [9], potentially augment PIC amplitudes, and modulate MU behavior by reducing recruitment thresholds of HTMU while increasing the firing rates of LTMUs [26], it is plausible that caffeine may improve the neural determinants of RFD. Such facilitation might be particularly evident in the recruitment rate of MUs at contraction onset, a critical determinant of early-phase RFD [129].

## 7. Training in a Caffeinated State

Caffeine’s ergogenic potential has been documented for over a century [39], with numerous studies reporting significant performance improvements across various exercise modalities and contexts [9,14,87,94,139]. As discussed in the previous sections, caffeine acutely enhances neural drive by increasing central excitability, thereby improving voluntary muscle activation [28]. Moreover, caffeine has been shown to reduce perceived effort and pain [71,80,82,86,88], ultimately allowing for greater total work output [71]. In light of this evidence, recent research has begun to explore whether repeated caffeine ingestion during training sessions may potentiate long-term neuromuscular adaptations to strength training.

The available evidence for chronic ergogenic effects of caffeine during training remains mixed. For instance, a 3 mg·kg^−1^ dose of caffeine enhanced one-repetition maximum (1RM) gains in both the bench press and back squat after six weeks of training compared with a control condition [140]. Using the same dosage, Giráldez-Costas et al. [141] reported greater velocity-based improvements in the Smith machine bench press after four weeks of training, although 1RM gains were comparable to the control group. This discrepancy may reflect differences in training protocols: Kemp et al. [140] trained participants to failure, a regimen more likely to elicit maximal strength improvements. In contrast, Tamilio et al. [142] and Pakulak et al. [143] found no significant differences in the magnitude of strength gains between caffeine and control groups in either upper- or lower-body exercises. Notably, Tamilio et al. [142] observed a significantly greater total workload per training session in the caffeine group. Given that caffeine does not appear to enhance post-exercise recovery and may even impair it under certain conditions [144], an attenuated recovery capacity could offset any training-session advantages and explain null effects on long-term strength.

Strength training has been extensively shown to induce adaptations within the neuromuscular system, particularly at the level of MUs [145,146,147]. Recent evidence suggests that MU adaptations following strength training are strongly mediated by improved modulation of neural drive, both in terms of magnitude and common drive stability [126,147,148]. Given caffeine’s capacity to modulate central command, MU behavior represents a compelling domain through which to investigate training-induced neuromuscular adaptations under chronic caffeine intake. To date, only one study has explored this topic [149]. The authors combined blood flow restriction (BFR) training with pre-exercise caffeine ingestion (6 mg·kg^−1^, administered one hour prior) over a four-week wrist extensor training period. Testing involved ramp contractions, during which Lin and colleagues observed enhanced force control in the descending phase (i.e., force release) and increased firing rate. Moreover, force signal complexity significantly improved, corresponding to reduced force fluctuations [149]. Signal complexity is a hallmark of healthy physiological systems, and its reduction typically reflects a diminished capacity to adapt to internal or external perturbations [150]. Caffeine has previously been shown to mitigate the loss of torque complexity [151] and to increase brain entropy [65]. Therefore, the findings of Lin et al. [149] support a centrally mediated ergogenic influence of caffeine even in the context of chronic training adaptations. However, since caffeine was administered in conjunction with BFR, the specific contribution of caffeine remains uncertain. Future studies isolating caffeine’s chronic effects are needed, particularly to examine MU parameters that may adapt differentially under its influence, such as PICs and common drive.

## 8. Conclusions

Caffeine has been extensively shown to facilitate neuromuscular performance. In vitro studies on animal models, however, appear to reject caffeine’s ability to act directly on human muscle tissue at physiological doses, although a potential stimulatory effect on myokine release cannot be excluded. Consequently, its ergogenic action is thought to be predominantly centrally mediated, primarily through the antagonism of adenosine receptors. This mechanism enhances cortical excitability and corticospinal communication, leading to improved MU recruitment and firing rate, and ultimately to more efficient voluntary movement execution. Although caffeine’s ergogenic effects benefit most individuals, considerable interindividual variability exists, largely attributable to genetic factors. Furthermore, limited evidence is available regarding the acute and chronic modulation of motor learning, MU adaptations, and sex-related differences in these parameters. Addressing these gaps will enhance understanding of caffeine’s role as an ergogenic aid for neuromuscular performance and support practitioners in tailoring supplementation strategies more effectively. Finally, it should be emphasized that much of the available evidence on the neural strategies underpinning movement execution, as well as the mechanistic interpretations discussed in this review, is derived from isometric contractions, a condition under which HDsEMG recordings are most reliable. Although these studies have provided valuable insights, there remains a clear need for more in-depth investigations of dynamic movements, which will likely become feasible as more accurate algorithms for extracting neural strategies from EMG signals during dynamic contractions are developed.

## Figures and Tables

**Table 1:** Sites of caffeine action and associated outcomes.

Level	Primary Target	Mechanism	Outcome
CNS	Adenosine A2A receptor (antagonism)	↑ Dopamine and norepinephrine, ↓ Cortical inhibition	↑ Cortical excitability, ↑ Arousal
Spinal	Monoaminergic input to motor neurons	↑ PIC amplitude, ↑ Excitatory drive	↑ Motor neuron output, ↑ H-reflex slope
Motor Unit	Supraspinal drive, MU recruitment thresholds	↓ RT, ↑ Firing rate (esp. in LTMU)	↑ RFD, improved torque steadiness
Peripheral Muscle	Sarcoplasmic Ca^2+^ release *, Na^+^/K^+^ pump	Improved excitation–contraction coupling *	↑ Contractile efficiency, Delayed Fatigue Onset
Systemic	Adrenal medulla, β-adrenergic activity	↑ Catecholamines, ↑ Lipolysis	↑ Energy availability, ↑ Alertness

* Only at supraphysiological doses (>70 µM); “↑” indicates an increase in the measured activity, release, or parameter, whereas “↓” indicates a decrease in the measured activity, release, or parameter; CNS = Central Nervous System; PIC = Persistent Inward Currents; RT = Recruitment Threshold; LTMU = Lower-threshold Motor Units; RFD = Rate of Force Development; MU = Motor Unit.

## Data Availability

No new data were created or analyzed in this study.

## Abbreviations

The following abbreviations are used in this manuscript:

CNS	Central Nervous System
CYP1A2	Cytochrome P450 1A2
SCL	Skin Conductance Level
EEG	Electroencephalography
fMRI	Functional Magnetic Resonance
CSP	Cortical Silent Period
MEP	Motor Evoked Potential
MRCP	Motor-related Cortical Potential
RPE	Rate of Perceived Exertion
BBB	Blood–Brain Barrier
LTP	Long-term Potentiation
H_max_	H-Reflex maximal
H_slp_	H-Reflex slope
CMEPs	Cervicomedullary Motor-evoked Potentials
PIC	Persistent Inward Currents
HDsEMG	High-density Surface Electromyography
MU	Motor Unit
RMS	Root Mean Square
MVF	Maximal Voluntary Force
HTMU	Higher-threshold Motor Unit
LTMU	Lower-threshold Motor Unit
RT	Recruitment Threshold
RFD	Rate of Force Development
1RM	1-Repetion Maximum
BFR	Blood Flow Restriction
